# Identification of the asymptomatic *Plasmodium falciparum* and *Plasmodium vivax* gametocyte reservoir under different transmission intensities

**DOI:** 10.1371/journal.pntd.0009672

**Published:** 2021-08-27

**Authors:** Cristian Koepfli, Wang Nguitragool, Anne Cristine Gomes de Almeida, Andrea Kuehn, Andreea Waltmann, Eline Kattenberg, Maria Ome-Kaius, Patricia Rarau, Thomas Obadia, James Kazura, Wuelton Monteiro, Andrew W. Darcy, Lyndes Wini, Quique Bassat, Ingrid Felger, Jetsumon Sattabongkot, Leanne J. Robinson, Marcus Lacerda, Ivo Mueller

**Affiliations:** 1 Population Health & Immunity Division, Walter & Eliza Hall Institute, Parkville, Australia; 2 Department of Medical Biology, University of Melbourne, Parkville, Australia; 3 University of Notre Dame, Eck Institute for Global Health, Department of Biological Sciences, Notre Dame, Indiana, United States of America; 4 Department of Molecular Tropical Medicine and Genetics, Faculty of Tropical Medicine, Mahidol University, Bangkok, Thailand; 5 Fundação de Medicina Tropical Dr. Heitor Vieira Dourado (FMT-HVD), Manaus, Brazil; 6 Universidade do Estado do Amazonas, Manaus, Brazil; 7 ISGlobal, Hospital Clínic—Universitat de Barcelona, Barcelona, Spain; 8 Papua New Guinea Institute of Medical Research, Madang, Papua New Guinea; 9 Hub de Bioinformatique et Biostatistique, Département Biologie Computationnelle, Institut Pasteur, Paris, France; 10 Unité Malaria: parasites et Hôtes, Département Parasites et Insectes Vecteurs, Institut Pasteur, Paris, France; 11 Centre for Global Health & Diseases, Case Western Reserve University, Cleveland, Ohio, United States of America; 12 National Health Training and Research Institute, Ministry of Health, Honiara, Solomon Islands; 13 Vector Borne Diseases Program, Ministry of Health, Honiara, Solomon Islands; 14 Centro de Investigação em Saúde de Manhiça (CISM), Maputo, Mozambique; 15 ICREA, Barcelona, Spain; 16 Pediatric Infectious Diseases Unit, Pediatrics Department, Hospital Sant Joan de Déu (University of Barcelona), Barcelona, Spain; 17 Consorcio de Investigación Biomédica en Red de Epidemiología y Salud Pública (CIBERESP), Madrid, Spain; 18 Swiss Tropical and Public Health Institute, Basel, Switzerland; 19 Mahidol Vivax Research Unit, Faculty of Tropical Medicine, Mahidol University, Bangkok, Thailand; Menzies School of Health Research, AUSTRALIA

## Abstract

**Background:**

Understanding epidemiological variables affecting gametocyte carriage and density is essential to design interventions that most effectively reduce malaria human-to-mosquito transmission.

**Methodology/Principal findings:**

*Plasmodium falciparum* and *P*. *vivax* parasites and gametocytes were quantified by qPCR and RT-qPCR assays using the same methodologies in 5 cross-sectional surveys involving 16,493 individuals in Brazil, Thailand, Papua New Guinea, and Solomon Islands. The proportion of infections with detectable gametocytes per survey ranged from 44–94% for *P*. *falciparum* and from 23–72% for *P*. *vivax*. Blood-stage parasite density was the most important predictor of the probability to detect gametocytes. In moderate transmission settings (prevalence by qPCR>5%), parasite density decreased with age and the majority of gametocyte carriers were children. In low transmission settings (prevalence<5%), >65% of gametocyte carriers were adults. Per survey, 37–100% of all individuals positive for gametocytes by RT-qPCR were positive by light microscopy for asexual stages or gametocytes (overall: *P*. *falciparum* 178/348, *P*. *vivax* 235/398).

**Conclusions/Significance:**

Interventions to reduce human-to-mosquito malaria transmission in moderate-high endemicity settings will have the greatest impact when children are targeted. In contrast, all age groups need to be included in control activities in low endemicity settings to achieve elimination. Detection of infections by light microscopy is a valuable tool to identify asymptomatic blood stage infections that likely contribute most to ongoing transmission at the time of sampling.

## Author summary

*Plasmodium vivax* and *Plasmodium falciparum* cause the vast majority of all human malaria cases. Across all transmission settings, a large proportion of infections of the two species remain asymptomatic. These infections are not diagnosed and treated by control programs focusing on clinical cases. They can carry gametocytes, the sexual stage of the parasite that establishes infections in mosquitos, thus asymptomatic infections contribute to transmission. In order to determine who is likely to contribute to transmission, gametocyte densities were measured by sensitive molecular methods in afebrile individuals in four countries. The proportion of infections with gametocytes varied greatly among surveys, and was higher in regions that had experienced low transmission for extended periods of time. In moderate-high transmission settings, gametocyte densities were particularly high in children below six years, highlighting the importance that interventions to reduce transmission include this age group. The majority of gametocyte carriers was positive by light microscopy. The comprehensive data on gametocyte carriage presented here lays the foundation for the development of more effective screen and treat activities to reduce malaria transmission.

## Introduction

A variety of malaria control interventions aim to reduce the transmission of parasites from the human to the mosquito host. Vector control tools such as bed nets and indoor residual spraying [1] lower the risk for infection in humans, and for onward transmission. Additional public health interventions primarily aimed at reducing human-to-mosquito transmission are currently being applied or developed, e.g. mass screening and treatment [2], mass drug administration [3], transmission blocking vaccines [4], and ivermectin administration [5].

Interventions that reduce human-to-mosquito transmission are most effective when they target individuals within a population who contribute most to transmission. Not all individuals with blood-stage parasitemia are equally infectious to mosquitos. Only a small fraction of all parasites in the human host develop into sexual stages termed ‘gametocytes’; parasite development in the mosquito relies on uptake and subsequent mating of male and female gametocytes [6].

Developing *P*. *falciparum* gametocytes are sequestered in extravascular sites such as bone marrow for 1–2 weeks [7, 8]. Gametocytes appear in the blood stream after the first wave of asexual parasites, and, in the case of symptomatic malaria cases, are detectable by microscopy often only 1–2 weeks after presentation with fever [9]. Mature gametocytes infective to mosquitos circulate in peripheral blood for a period of a few days to up to three weeks [10–12]. Most commonly used antimalarials used to treat symptomatic cases do not clear sequestered or mature gametocytes. This is also the case for artemisinin combination therapy (ACT), the first-line drug in most *P*. *falciparum* endemic countries. As a result of the continued release of sequestered gametocytes after treatment, and their circulation for days to weeks, individuals can carry gametocytes for several weeks after treatment [12–14]. Primaquine is the only approved drug that clears *P*. *falciparum* gametocytes. Low-dose primaquine has been shown to reduce duration of gametocytemia after treatment [15, 16].

*P*. *vivax* gametocytes infective for mosquitoes appear within 2–3 days after blood-stage asexual parasitemia commences and might circulate for only 2–3 days [17, 18]. Gametocytes are present in the majority of clinical cases [19–21]. *P*. *vivax* gametocytes are sensitive to common drugs such as chloroquine or artemether-lumefantrine. In drug trials, up to 90% of patients were gametocyte-free one day after treatment, and virtually none carried gametocytes at day 7 [20–22].

In most malaria endemic regions the vast majority of infections are asymptomatic, i.e. not associated with fever. 50–80% of infections are not detectable by microscopic inspection of blood smears [23, 24]. Since gametocytes account for only a small proportion of all parasites in peripheral blood, they are more difficult to detect by light microscopy (LM) than asexual parasites. The development of molecular methods to detect gametocyte-specific RNA transcripts by nucleic acid sequence-based amplification (NASBA) or reverse-transcriptase quantitative PCR (RT-qPCR) has allowed for detection of submicroscopic gametocytemia [25, 26]. However, few studies have reported gametocyte carriage in asymptomatic individuals in non-African settings, especially with respect to *P*. *vivax* [27–33]. It is not known whether gametocyte densities differ across regions of different transmission intensities, and the distribution of gametocyte carriers among various demographic groups within a community is not well understood. Knowledge of these epidemiological variables is needed to target transmission-reducing interventions to those at highest risk of gametocyte carriage and to understand the long-term impact of these interventions on progress towards malaria elimination.

To advance our understanding of *P*. *falciparum* and *P*. *vivax* gametocyte carriage across a range of transmission settings, five cross-sectional surveys involving a total of 16,493 individuals were conducted in Brazil, Thailand, Papua New Guinea (PNG) and Solomon Islands [27–31]. The surveys included endemic areas where transmission was moderate to high or had recently decreased (PNG in 2010 and 2014, Solomon Islands) and regions where transmission was low, with *P*. *vivax* being the predominant parasite (Brazil, Thailand). Blood samples were collected from the general population irrespective of symptoms of malaria illness. Total blood-stage parasites and gametocytes were quantified by LM and sensitive qPCR and RT-qPCR assays using the same methodology across all studies, allowing for the first direct comparison across transmission intensities.

## Study sites and methods

### Ethics statement

The study was approved by the PNG Institute of Medical Research IRB (1116/1204), the PNG Medical Research Advisory Committee (MRAC numbers 11.21, 05.20, 12.06, 12.01), the WEHI Human Research Ethics Committee (WEHI HREC, 12/09), the Case Western Reserve University Hospitals of Cleveland Medical Center (CWRU UHCMC, 05-11-11), the Solomon Islands National Health Research Ethics Committee (HRC12/022), the Ethics Committee of the Faculty of Tropical Medicine, Mahidol University, Thailand (EC approval number MUTM 2012-044-01), the Brazilian National Committee of Ethics (CONEP) (349.211/2013), and by the Committee of Ethics for Clinical Investigation from Barcelona Hospital Clinic (7306/2012). Prior to sample collection, the aims of the study were explained to all individuals and informed written consent was obtained from participants or, in case of minors, from their guardians.

### Study sites and sample collection

Details on study sites are given in Table 1. Community sensitization took place 1–2 weeks prior to sample collection. Convenience sampling was applied to select households for the surveys. All members of the selected households were noted on a list, and individuals above 6 months of age were invited to participate. Sampling started in the morning and continued throughout the day. As children might be in school during this time and adults away for work, efforts were made to sample school-aged children and working adults after they returned home. The age and gender distributions of the study participants for each survey is given in S1 Table, and is expected to be representative for the population. Overall, the distributions were similar across surveys, with the exception of a lower proportion of all sampled being small children in Brazil and Thailand.

**Table 1:** Cross-sectional surveys included in this study.

Survey Location	Year	Individuals	Measures of recent malaria transmission	Reference
PNG, Madang Province	2010	2083	Prevalence by PCR 41.7% *P*. *vivax* and 42.1% *P*. *falciparum* in 2006. After bed net roll out in 2007–2008, 4–5 fold decrease of clinical incidence in children, and 3-fold decrease of *P*. *vivax* molecular force of infection.	[27, 28]
	2014	2517		
Solomon Islands, Ngella, Central Islands Province	2012	3501	10-fold decline in clinical incidence in last 20 years across Solomon Islands. In Tetere, Guadalcanal Province, prevalence by microscopy was 12.9% *P*. *falciparum* and 19.1% *P*. *vivax* in 2004–2005.	[30]
Thailand, Kanchanburi and Ratchaburi Provinces	2013	4309	Prevalence by LM 0.1–5.2% *P*. *falciparum* and 0.2–5.9% *P*. *vivax* in 2000–2004	[29]
Brazil, Manaus	2012–2013	4083	Prevalence by PCR <1–2% *P*. *falciparum* and 8.5–15% *P*. *vivax* in different regions in the Amazon in 2002–2008. Since then 10-fold decrease in prevalence, virtually no *P*. *falciparum* left.	[31]

The study sites experience little or moderate seasonality in transmission. In PNG, the rainy season is from December to April. Both surveys were conducted in May to July, i.e. after the rainy season. There is minimal seasonal variation in Solomon Islands; samples were collected in May to June. In Thailand, the peak transmission season is from April to July, and samples were collected in September and October. In Manaus, Brazil, highest incidence occurs from May to September. Half of the samples were collected in September to early January, and the other half in August to September.

Form each participant, 250 μL blood was collected by finger prick into 2 mL EDTA microtainers (BD). Hemoglobin levels were determined using the HemoCue handheld meter. For RNA extraction and gametocyte detection, 50 μL blood was transferred into tubes containing 250 μL of RNAprotect (Qiagen) in the field. Samples in RNAprotect were kept on ice packs in the field and transferred to -80°C storage every evening and kept there until RNA extraction. The remaining 200 μL blood were kept in the EDTA microtainer, also kept on ice packs, and transferred to -20°C storage until DNA extraction.

### Molecular methods

DNA was extracted from 200 μL whole blood kept in EDTA microtainers and eluted in 200 μL elution buffer. Parasites were quantified by qPCR using the 18S rRNA gene as target [34]. The assay used detects one copy of the gene. 4 μL DNA was screened by qPCR, thus the limit of detection was 0.25 parasites/μL blood (i.e. 1 genome per 4 μL DNA). RNA was extracted from 50 μL whole blood kept in RNAprotect, and eluted in 50 μL elution buffer. 2 μL RNA was screened by RT-qPCR. Gametocytes were quantified by RT-qPCR of the female gametocyte-specific transcripts *pfs25* and *pvs25* [35].

Procedures for sample collection, qPCR, and RT-qPCR were standardized between all sites. Results of individual cross-sectional surveys have been published previously [28–31, 35]. For qPCR and RT-qPCR, standardized plasmids were distributed to all laboratories and run along samples for relative quantification and estimation of sensitivity. Sensitivity of all assays was 0.5–1 copies/uL DNA or RNA. For absolute measurements of copy numbers, a subset of samples from each laboratory was quantified by droplet digital PCR [36]. Due to different procedures of RNA sample collection in Solomon Islands, only positivity, but not *pvs25* and *pfs25* copy numbers, were included in the analysis. Expert microscopy was conducted in PNG, Solomon Islands, and for parts of the survey in Brazil. To determine multiplicity of infection (MOI), *P*. *falciparum* infections were genotyped by *msp2* [37], and *P*. *vivax* infections were genotyped by *msp1F3* and MS2 [38].

### Data analysis

The following definitions are used: ‘proportion gametocyte-positive infections’ describes the number of gametocyte carriers divided by the number infected with asexual parasites and/or gametocytes; ‘population gametocyte prevalence’ is the prevalence of gametocytes among all individuals, infected and non-infected [39].

Multivariable regression models were used to predict factors associated with the proportion gametocyte-positive infections and gametocyte density. Parasite densities were log_10_ transformed for all calculations. To correct for imperfect detection of gametocytes and include low-density infections without gametocytes detected in multivariable models, +0.1 was added to all gametocyte density values prior to log_10_-tranformation. For multivariable analyses, individuals were grouped into age classes ≤6, >6–12, >12–20, and >20 years. Survey and age group were included as fixed effects. The ratio of *pfs25* or *pvs25* transcripts per parasite genome was assessed, representing the proportion of gametocytes among all parasites. Infections (*Pf*: n = 12, *Pv*: n = 5) with densities at the technical limit of detection of 0.25 copies/μL blood (i.e. 1 DNA/RNA template per PCR) were excluded from correlation analysis as quantification is imprecise at very low densities, and including them at a set density of 0.25 copies/μL would artificially increase correlation. All data is available in supplementary file S1 Data.

## Results

### Prevalence and parasite density

**Fig 1:**
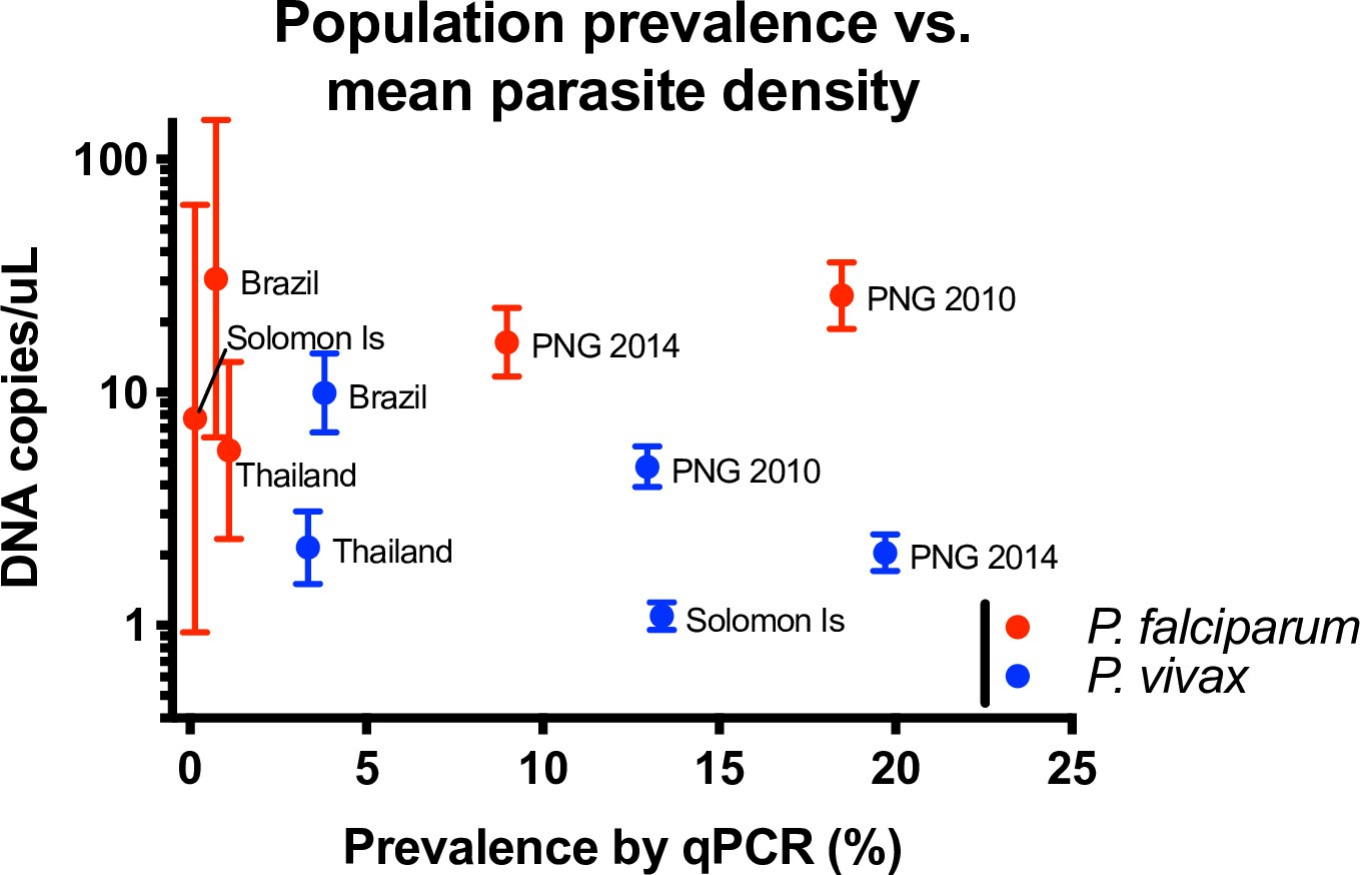
Parasite prevalence and mean parasite density by qPCR in five cross-sectional surveys. Error bars represent 95% confidence intervals.

Prevalence of *P*. *falciparum* by qPCR ranged from 0.14% (Solomon Islands, 5/3501) to 18.5% (PNG 2010, 385/2083), and *P*. *vivax* prevalence from 3.3% (Thailand, 144/4309) to 19.7% (PNG 2014, 496/2517) (Table 2 and Fig 1). Light microscopy (LM) detected 27.9–80.0% of *P*. *falciparum*, and 13.3–51.8% of *P*. *vivax* infections positive by qPCR (Table 2). Mean parasite densities of both species differed significantly between surveys (one-way ANOVA *P*<0.0001), with no clear trend between population parasite prevalence and mean parasite densities (Table 2 and Fig 1). In each survey, 80.0–94.5% of all *P*. *falciparum* infections and 80.0–97.5% of all *P*. *vivax* infections (by qPCR) were asymptomatic, i.e., not accompanied by measured or reported febrile illness in the preceding 48 hours.

**Table 2:** Parasite and gametocyte prevalence by LM and qPCR and RT-qPCR, and parasite densities by qPCR.

	PNG 2010	PNG 2014	Solomon Is.	Thailand	Brazil
Number of participants	2083	2517	3501	4309	4083
***P*. *falciparum***					
Prevalence by LM^1^	7.44% (155/2083)	2.74% (69/2513)	0.11% (4/3501)	NA	0.55% (11/2010)
Prevalence by PCR	18.48% (385/2083)	8.98% (226/2517)	0.14% (5/3501)	1.11% (48/4309)	0.39% (16/4083)
qPCR copies/uL [CI95]	26.0 [18.7–36.0]	16.4 [11.4–23.0]	7.7 [0.9–63.8]	5.6 [2.4–13.5]	30.7 [6.4–147.1]
Proportion LM positive	37.40% (144/385)	27.88% (63/226)	80.00% (4/5)	NA	63.64% (7/11)
Infected + febrile/reported fever	12.77% (48/376)	5.48% (12/219)	20.0% (1/5)	8.7% (4/46)	16.67% (2/12)
Gametocyte prevalence by RT-qPCR	11.19% (233/2083)	3.85% (97/2517)	0.11% (4/3501)	0.86% (37/4309)	0.37% (15/4083)
Proportion gametocyte positive^2^	60.52% (233/385)	42.92% (97/226)	80% (4/5)	70.83% (34/48)	93.75% (15/16)
Proportion gametocyte carriers LM positive^3^	54.51%	37.11%	100.00%	NA	78.57%
***P*. *vivax***					
Prevalence by LM^1^	7.00% (146/2083)	2.70% (68/2513)	3.63% (127/3501)	NA	1.79% (36/2010)
Prevalence by PCR	12.96% (270/2083)	19.71% (496/2517)	13.37% (468/3501)	3.34% (144/4309)	3.82% (156/4083)
qPCR copies/uL [CI95]	4.8 [3.98–5.9]	2.0 [1.7–2.4]	1.1 [1.0–1.3]	2.2 [1.5–3.1]	10.0 [6.7–14.7]
Proportion LM positive	51.85% (140/270)	13.31% (66/496)	27.14% (127/468)	NA	37.21% (32/86)
Infected + febrile/reported fever	8.27% (22/266)	2.52% (12/477)	20.0% (93/465)	8.51 (12/141)	17.42% (27/155)
Gametocyte prevalence by RT-qPCR	6.34% (132/2083)	4.45% (112/2517)	3.14% (110/3501)	2.39% (103/4309)	2.03% (83/4083)
Proportion gametocyte positive^2^	48.89% (132/270)	22.58% (112/496)	23.5% (110/468)	71.53% (103/144)	53.21% (83/156)
Proportion gametocyte carriers LM positive^3^	84.09%	39.64%	49.09%	NA	58.14%
***P*. *falciparum*/*P*. *vivax* co-infections** ^4^					
Prevalence by LM	0.72% (15/2083)	0.32% (8/2513)	0% (0/3501)	NA	0% (0/2010)
Prevalence by qPCR	3.90% (81/2083)	1.63% (41/2517)	0.14% (5/3501)	0.26% (11/4309)	0.02% (1/4083)

NA = not available

^1^ Positive by LM for asexuals and/or gametocytes. In Brazil, LM was conducted only on a subset of n = 2010 samples.

^2^ Proportion of qPCR positive samples with gametocytes detected by RT-qPCR

^3^ Proportion of study subjects positive for gametocytes by RT-qPCR with asexual parasites and/or gametocytes detected by LM

^4^ Data in the Table for *P*. *falciparum* and *P*. *vivax* includes co-infections with the respective other species. Prevalence of co-infections represents co-infections among all individuals sampled (not among those positive by either species).

### Gametocyte carriage

**Fig 2:**
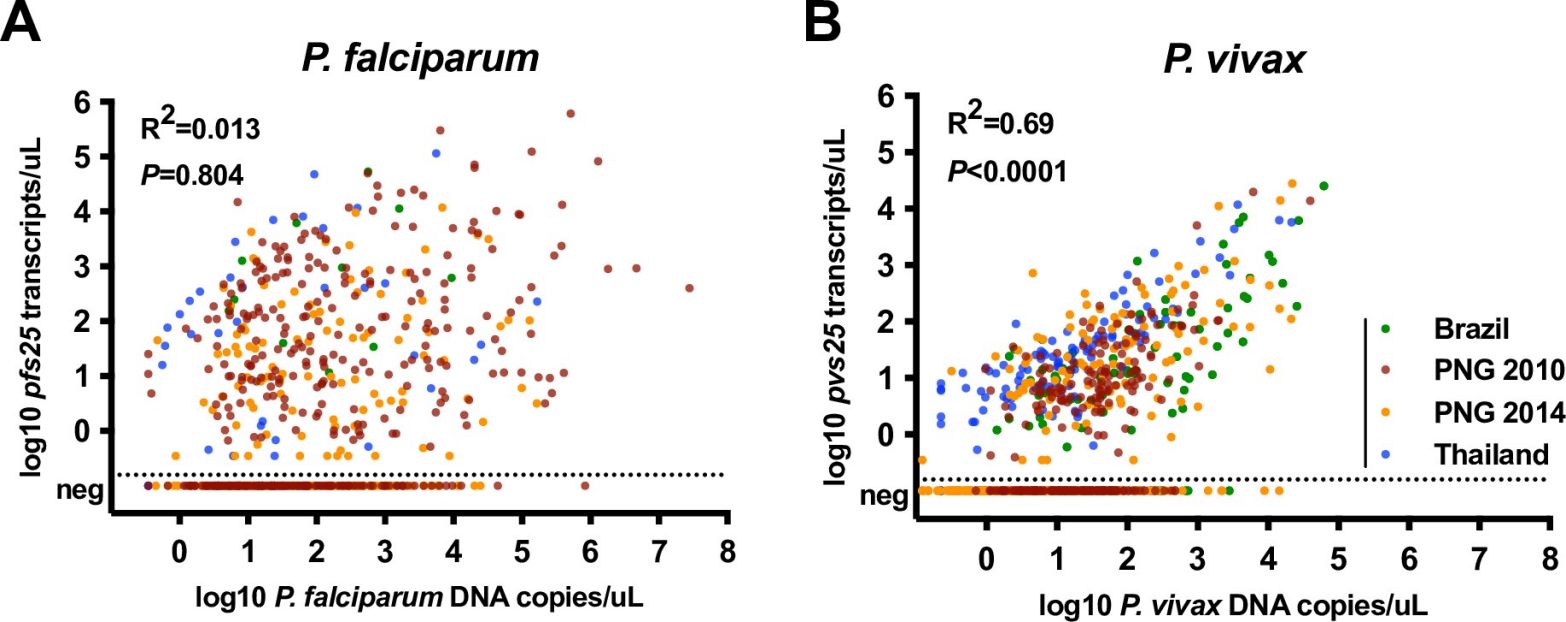
Relationship of *P*. *falciparum* (A) and *P*. *vivax* (B) parasite density by qPCR and *pfs25*/*pvs25* transcript numbers by RT-qPCR.

By *pfs25* RT-qPCR, the proportion of gametocyte-positive infections differed significantly between surveys (Chi-Square test, *P*<0.001, Table 2). 93.75% (15/16), 80.0% (4/5), and 70.8% (34/48) of *P*. *falciparum* qPCR-positive individuals carried gametocytes in Brazil, Solomon Islands and Thailand, respectively, and 42.9% and 60.5% in PNG 2014 and PNG 2010, respectively. This resulted in a population *P*. *falciparum* gametocyte prevalence ranging from 0.11% in Solomon Islands to 11.2% in PNG 2010 (Table 2). In univariate analysis across all surveys, no correlation between *P*. *falciparum* DNA copy number and *pfs25* copy numbers was observed (gametocyte-positive infections above technical limit of detection included, n = 379, R^2^ = 0.013, *P* = 0.804, Fig 2A). However, each 10-fold increase in DNA copy number increased the odds to detect *P*. *falciparum* gametocytes 1.59-fold (all infections included, n = 676, *P*<0.001).

By *pvs25* RT-qPCR, *P*. *vivax* gametocytes were detected in 22.6% and 23.5% of infected individuals in PNG 2014 and Solomon Islands, and in 48.9%, 53.7%, and 71.5% of infections in PNG 2010, Brazil, and Thailand (*P*<0.001, Table 2). This resulted in a population *P*. *vivax* gametocyte prevalence of 2.0–4.3%. *P*. *vivax* parasite and gametocyte densities were strongly correlated (n = 415, R^2^ = 0.69, *P*<0.0001, Fig 2B), and a 10-fold increase in DNA copy number increased the odds of detecting *P*. *vivax* gametocyte by 2.91-fold (n = 1501, *P*<0.001).

**Fig 3:**
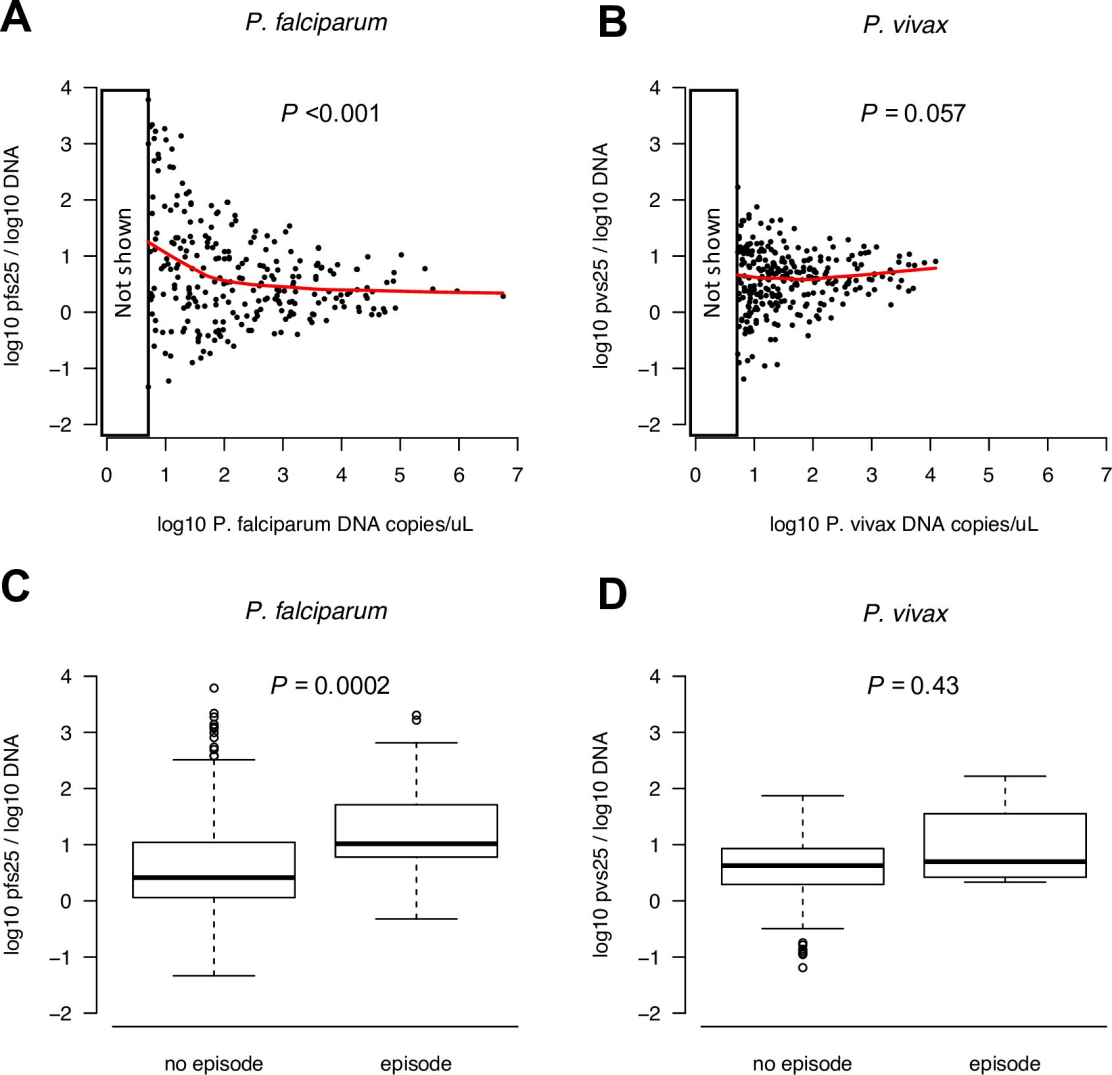
Proportion of gametocytes among all blood-stage parasites. Proportion *P*. *falciparum* (A) and *P*. *vivax* (B) gametocytes among all blood-stage parasites by parasite density with LOWESS splines (red). Very low-density infections with <5 DNA copies/uL are excluded, as quantification (and thus the calculated proportions) might be inaccurate at low densities. Panels C and D show transcript vs. total parasite density stratified into those infections with and without a febrile episode in the 2 weeks prior to sampling (self-reported febrile illness or antimalarial treatment). Boxplots show median (solid line), 25th and 75th percentile (box), whiskers extend up to 1.5-fold the interquartile range.

To assess whether the proportion of gametocytes among all blood-stage parasites differs between infections of different parasite density, the number of *pfs25* or *pvs25* transcripts per *P*. *falciparum* or *P*. *vivax* genome among gametocyte-positive samples was plotted (Fig 3A and 3B, infections with ≤5 DNA copies excluded). For *P*. *falciparum*, a 0.39-fold decrease in the proportion gametocytes per 10-fold increase in parasite density was observed (n = 255, *P*<0.001), while no significant change was observed for *P*. *vivax* (n = 243, 1.19-fold increase per 10-fold increase in parasite density, *P* = 0.057). A febrile episode and/or antimalarial treatment in the preceding 2 weeks increased the proportion gametocytes significantly for *P*. *falciparum* (n = 255, 27 episodes, Wilcoxon rank sum test *P* = 0.0002, Fig 3C), but had no impact on *P*. *vivax* gametocyte proportions (n = 288, 4 episodes, *P* = 0.4319, Fig 3D).

### Multivariate risk factors of gametocyte positivity and density

In multivariate analysis across all surveys, parasite density was a strong predictor for the probability that a sample was gametocyte-positive (Table 3). Each 10-fold increase in *P*. *falciparum* parasite density resulted in a 1.59-fold increase in the odds of gametocyte positivity, and in a 1.9-fold increase in gametocyte densities (Table 3). The correlation was much stronger for *P*. *vivax*, where each 10-fold increase in parasite density resulted in a 3.15-fold increase in the odds of gametocyte positivity and a 3.9-fold increase in gametocyte density (Table 3). Concordance between *P*. *vivax* genome and gametocyte density was even higher for infections above 5 DNA copies/uL (n = 373), with 9.0-fold more gametocytes per 10-fold increase in genome density.

**Table 3:** Multivariate predictors of gametocyte positivity and density.

**Gametocyte positivity**				
	*P*. *falciparum* (n = 647)		*P*. *vivax* (n = 1501)	
	OR	*P*	OR	*P*
log_10_ Pf/Pv copies	1.59	<0.0001	3.15	<0.0001
PNG 2010 (reference)	[Reference]		[Reference]	
PNG 2014	0.55		0.40	
Solomon Is.	8.54	<0.0001	0.62	<0.0001
Thailand	2.99		5.60	
Brazil	11.03		0.65	
age: 0–6 y (reference)	[Reference]			
age: >6–12 y	1.13			
age: >12–20 y	0.56	0.0006		
age: >20 y	0.47			
*P*. *vivax* PCR positive	0.41	<0.0001		
Reported malaria last 2 weeks	2.28	0.032		
**Gametocyte density**				
	*P*. *falciparum* (n = 642)		*P*. *vivax* (n = 1041)	
	Coef.	*P*	Coef.	*P*
log_10_ Pf/Pv copies	0.20	<0.0001	0.59	<0.0001
PNG 2010	[Reference]		[Reference]	
PNG 2014	-0.44		-0.08	
Thailand	0.58	<0.0001	0.83	<0.0001
Brazil	1.73		0.02	
age: 0–6 y (reference)	[Reference]		[Reference]	
age: >6–12 y	-0.33		-0.07	
age: >12–20 y	-0.76	<0.0001	-0.18	0.0167
age: >20 y	-0.82		-0.20	
*P*. *vivax* PCR positive	-0.51	<0.0001		
Reported malaria last 2 weeks	0.82	<0.0001		

(A) Gametocyte positivity, (B) gametocyte density. Additional factors tested, but not found to be associated, included fever measured at the day of survey or reported from the past 2 days, and reported malaria or antimalarial treatment in the past 2 weeks. In each subgroup, only variables with a significant effect (*P*<0.05) on gametocyte positivity or densities are shown.

Among *P*. *falciparum* positive individuals, the odds to detect gametocytes was 54% lower (*P*<0.0001) and gametocyte densities were 69% lower (*P*<0.0001) in individuals co-infected with *P*. *vivax*. Reported malaria or anti-malarial treatment in the past 2 weeks was associated with higher *P*. *falciparum* gametocyte prevalence and densities (Table 3). Parasite densities strongly decreased with increasing age. Even when including parasite density as confounder, *P*. *falciparum* gametocyte positivity and density decreased with increasing age, i.e., gametocyte densities decrease to a greater extent than blood-stage parasitemia (Table 3). Among *P*. *vivax* positive individuals, gametocyte densities, but not positivity decreased with age (Table 3). Apart from DNA copy numbers, no other significant associations were observed.

Factors affecting gametocyte density were assessed independently in PNG 2010 (moderate-high transmission), PNG 2014 (recently decreased transmission), and Thailand and Brazil pooled (long-time low transmission) (Table 4). For PNG 2010 results were very similar to pooled data from all surveys. In PNG 2014, for *P*. *falciparum*, gametocyte densities decreased with age and were lower in individuals co-infected with *P*. *vivax*, but were not significantly associated with parasite density (*P* = 0.085). In Thailand and Brazil, for both species, and for *P*. *vivax* in PNG 2014, no other factors than parasite density were significantly associated with gametocyte density.

**Table 4:** Mutlivarable predictors of *P*. *falciparum* and *P*. *vivax* gametocyte density under different transmission scenarios.

**A) *P*. *falciparum***						
	PNG 2010 (n = 364)		PNG 2014 (n = 226)		Thailand + Brazil (n = 59)	
	Coef.	*P*	Coef.	*P*	Coef.	*P*
log_10_ Pf copies	0.34	<0.001			0.37	0.015
age: 0–6 y (reference)	[Reference]		[Reference]			
age: >6–12 y	-0.37		-0.42			
age: >12–20 y	-0.71	0.002	-1.01	<0.001		
age: >20 y	-0.84		-1.03			
Pv PCR positive	-0.51	0.002	-0.81	<0.001		
Reported malaria last 2 weeks	1.13	<0.001				
**B) *P*. *vivax***						
	PNG 2010 (n = 252)		PNG 2014 (n = 496)		Thailand + Brazil (n = 300)	
	Coef.	*P*	Coef.	*P*	Coef,	*P*
log_10_ Pv copies	0.63	<0.001	0.47	<0.001	0.62	<0.001
age: 0–6 y (reference)	[Reference]					
age: >6–12 y	-0.25					
age: >12–20 y	-0.42	0.0009				
age: >20 y	-0.52					
Pf PCR positive						
Reported malaria last 2 weeks						

Mutlivarable predictors of *P*. *falciparum* and *P*. *vivax* gametocyte density in PNG 2010 (intermediate transmission), PNG 2014 (recent reduction in transmission), and Thailand and Brazil pooled (extended period of low transmission). In each subgroup, only variables with a significant effect (*P*<0.05) on gametocyte densities were included.

**Fig 4:**
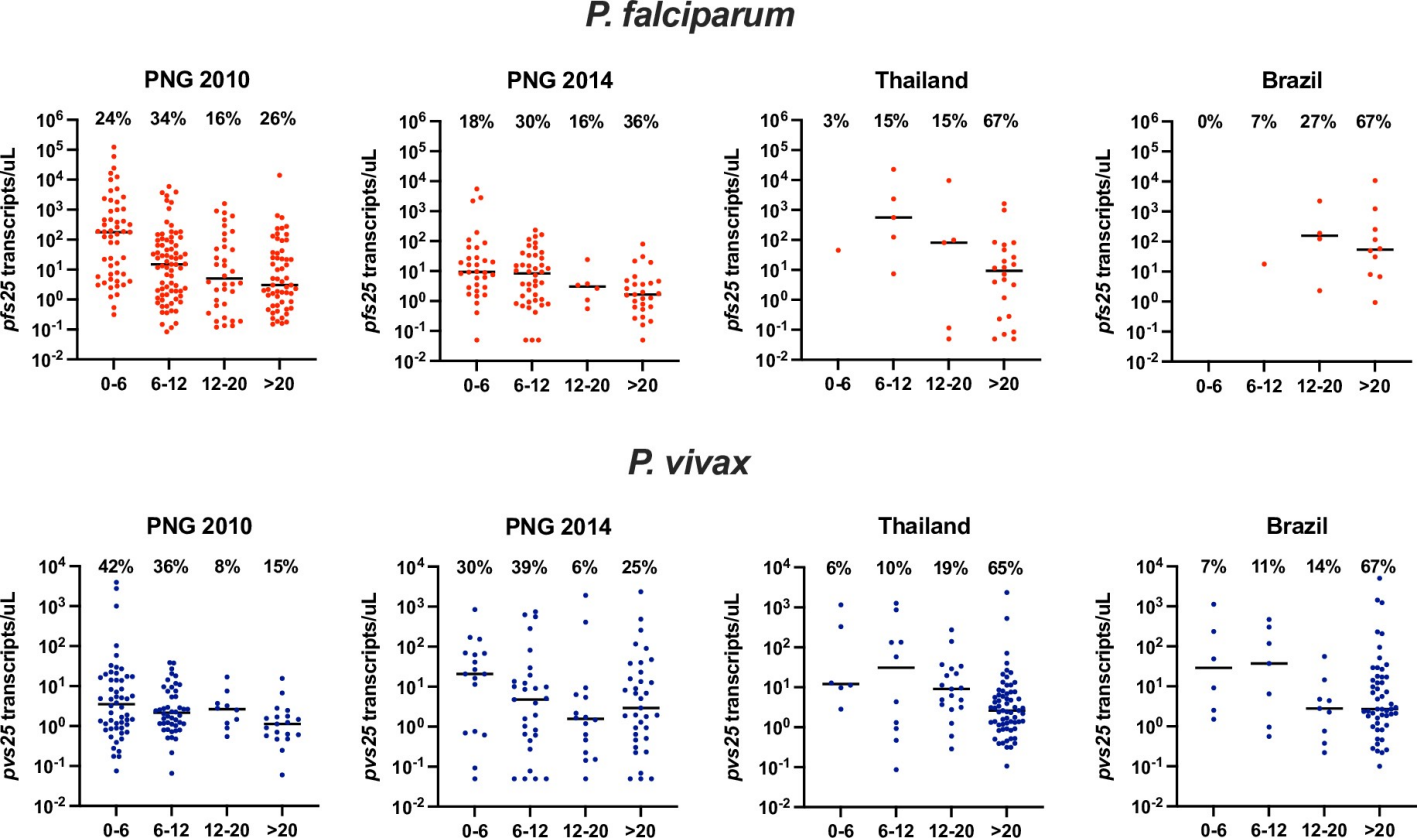
*pfs25* and *pvs25* transcript densities for each survey stratified by age group. The black line shows the median. The percentage shows the proportion of gametocyte carriers in an age group among all gametocyte carriers within this survey.

As a result of the correlation of parasite and gametocyte density and the decreasing parasite densities with age in both PNG surveys, for both species the majority of individuals with detectable gametocytes were children. 48–78% of gametocyte carriers were <12 years (Fig 4). In contrast, 65–67% of gametocyte carriers were >20 years in Thailand and Brazil.

When including parasite density in multivariable analysis to correct for differences in mean density among surveys, the proportion gametocyte positive infections, and gametocyte densities of both species differed significantly between surveys (*P*<0.0001, Table 3). The probability to detect gametocytes increased in all surveys with increasing genome density. An interaction analysis did not reveal a significant difference of this increase between surveys (*Pf*: n = 676, *P* = 0.471; *Pv*: n = 1501, *P* = 0.512). Likewise, *P*. *falciparum* and *P*. *vivax* gametocyte densities were not affected by an interaction between DNA copies and survey (*Pf*: n = 375, *P* = 0.877, *Pv*: n = 415, *P* = 0.132).

Hemoglobin measurements and multiplicity of infection data were available from the surveys in PNG and Solomon Islands; neither affected gametocyte positivity or density in multivariate analysis (S2 and S3 Tables).

### Ability of light microscopy to diagnose gametocyte carriers

Restricting the analysis to sites where LM was conducted (PNG, Solomon Islands, Brazil, n = 10,107), *P*. *falciparum* gametocytes were detected by RT-qPCR in 80.2% (178/222) of LM-positive individuals. Among LM-negative individuals, gametocytes were detected in 40.2% (170/422) of individuals. *Pfs25* transcript densities were nearly 10-fold higher in LM-positive individuals (33.40 vs. 4.27 transcripts/μL, *P* = 0.001). The ability of LM to detect *P*. *falciparum* gametocyte carriers differed significantly between surveys (*P*<0.001, Table 2), and appeared to be particularly high when prevalence was low. For example, 4/4, and 7/10 *P*. *falciparum* gametocyte carriers were LM-positive in Solomon Islands and Brazil, respectively (Table 2).

*P*. *vivax* gametocytes were detected by RT-qPCR in 64.9% (235/362) of LM-positive individuals, but in only 16.7% (163/976) of LM-negative individuals. Mean gametocyte densities in LM-positive individuals were almost twice as high compared to LM-negative individuals (6.02 vs. 3.44 transcripts/μL, *P*<0.001). Thus, approximately 50% of *P*. *falciparum* and 59% of *P*. *vivax* gametocyte carriers were positive by light microscopy.

## Discussion

We observed substantial differences in the proportion of *P*. *falciparum* and *P*. *vivax* infections carrying detectable gametocytes in 5 cross-sectional surveys representing distinct malaria-epidemiological contexts. The proportion of gametocyte-positive infections in community surveys is heavily impacted by the sensitivity of the assays used for parasite and gametocyte detection [39]. The use of the identical methodology and external reference standards for all surveys allowed, for the first time, direct comparisons between regions of different transmission intensity.

Blood-stage parasite densities were a strong predictor for gametocyte positivity and could largely explain differences between surveys. In most surveys, the majority of gametocyte carriers of both *Plasmodium* species (as determined by RT-qPCR) were positive by LM for asexual parasites. A lower proportion of gametocyte carriers was LM-positive in Madang 2014 (both species) and Solomon Islands (*P*. *vivax*), where transmission had declined in the years prior to the surveys [28, 30], and parasite densities were very low. In these surveys, a large proportion of gametocyte carriers could not be diagnosed by microscopy. In contrast, in the sites where transmission had been reduced for longer (Brazil, *P*. *falciparum* in Solomon Islands), expert LM or other tools such as RDT remain sufficiently sensitive to identify the majority of gametocyte carriers.

Pronounced age trends in gametocyte carriage were evident in PNG and Solomon Islands. Prevalence of infection peaked in children or adolescents, and parasite densities decreased rapidly with increasing age, most likely due to the acquisition of immunity. As a result, the vast majority of gametocytes were detected in children below 6 years, especially for *P*. *vivax*. This contrasts findings from *P*. *falciparum* in Africa, where school-age children were proposed to contribute most to transmission densities [40, 41]. In moderate-high transmission settings and in regions of steep decline in transmission in recent years, gametocyte densities decreased even faster than parasite densities with age. Thus, changes in parasite prevalence and density with age might not appropriately reflect changes in transmission potential. In Brazil and Thailand, the risk of infection increased with increasing age, age trends of parasite densities were moderate, and as a result no age trends in gametocyte densities were evident.

Apart from parasite density, limited effects of transient factors on gametocyte densities were observed. Multiple clone infection or hemoglobin levels did not affect gametocyte carriage of either species. For *P*. *vivax*, a constant proportion of gametocytes among all parasites was observed irrespective of parasite density. In the case of *P*. *falciparum*, high proportions of gametocytes were observed in a subset of infections with low-to-moderate densities The 2-week sequestration of developing *P*. *falciparum* gametocytes results in a temporal lag of peak gametocytemia following peak parasitemia [6]. Thus, infections with low parasite but high gametocyte densities might have experienced a recent wave of asexual parasitemia [42]. This is corroborated by the fact that self-reported febrile illness in the two weeks prior to sample collection resulted in higher gametocyte densities (Fig 3C and 3D). Conversion of a large proportion of all parasites into gametocytes when parasite densities drop to very low levels has also been described in a rodent malaria model [43]. Longitudinal studies with frequent sampling will be needed to assess how closely *P*. *falciparum* gametocyte density reflects parasite density in the preceding 2 weeks.

As an exception to the limited impact of transient factors, a lower proportion *P*. *falciparum* gametocyte positive infections and lower gametocyte densities were observed in individuals co-infected with *P*. *vivax*. It is not known whether co-infection results in an adjustment of the gametocyte conversion rate, or whether multi-species infection is a surrogate marker for higher exposure, and thus, higher levels of gametocyte-specific immunity.

**Fig 5:**
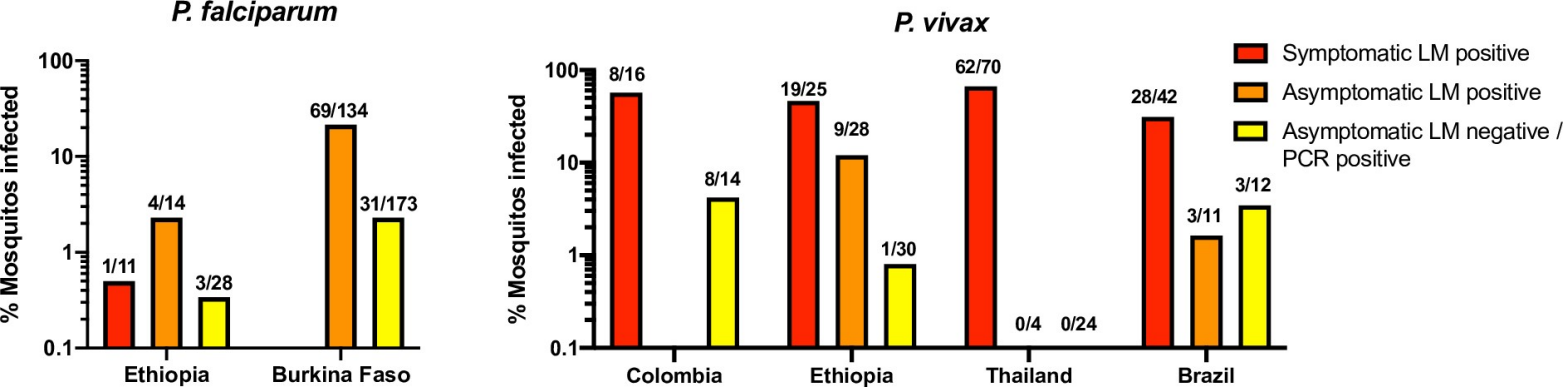
Infectivity of *P*. *falciparum* and *P*. *vivax* infections in membrane-feeding assays based on clinical status and ability of light microscopy to diagnose infections. ‘% Mosquitos infected’ shows the combined proportion of mosquitos infected including individuals that did not infect any mosquitos. n/N show the proportion of individuals that infected at least one mosquito. Infectivity varied widely, but with the exception of the study on *P*. *vivax* in Brazil, microscopy-positive individuals were 5-10-fold more infective that those with submicroscopic infection. Data from [45, 47–50].

Mosquito feeding assays have repeatedly shown a correlation between parasite density and infectivity. Few studies have included asymptomatic individuals and individuals negative by LM for gametocytes and asexual stages [44–50]. With few exceptions, e.g., one study on *P*. *vivax* infectivity in Brazil [48], individuals with either asexual parasites or gametocytes detected by LM were far more infective than submicroscopic infections (Fig 5). For *P*. *vivax*, in Thailand a steep increase in infectivity was found at densities (by LM) of 10–100 parasites/uL, with little effect if densities increased further [47]. This density closely matches the limit of detection of expert LM. The finding in the present study of a majority of gametocytes concentrated in LM-positive individuals, together with the results from mosquito-feeding studies, corroborate that LM-positive individuals likely are the main infectious reservoir.

Studies assessing gametocyte densities and infectivity over time will be required to determine what proportion of low-density infections will rise in density and become highly infective. The importance of male gametocyte densities to predict infectivity is increasingly recognized. In low density infections, male gametocytes might be the limiting factor for onward transmission [51]. Measuring male gametocyte densities in addition to female densities, as measured by *pfs25* and *pvs25* RT-qPCR, is expected to allow for better predictions of infectivity [51]. Due to the non-linear relationship between gametocyte density and infectivity, age trends in gametocyte density might not fully reflect infectivity. Lastly, while higher gametocyte densities result in increased oocyst numbers in mosquitos [51], it is unclear whether oocyst numbers have an effect on the efficiency of onward transmission.

Even after correcting for different mean parasite densities between surveys, a higher proportion gametocyte positive samples were found for both species in Thailand and for *P*. *falciparum* in Brazil and Solomon Islands compared to PNG. Recent malaria control activities have resulted in significant changes in vector composition and biting behavior in the study sites [52, 53]. A reduction in the number of mosquito bites or shift towards a less competent vector might select for parasites with higher gametocyte conversion rates. Such a selection has been suggested by recent genome and transcriptome studies. Expression of the AP2-G transcription factor and additional epigenetic factors involved in gametocytogenesis were adjusted to transmission levels in *P*. *falciparum* populations in East Africa [54], and the *gametocyte development gene 1* (*gdv1*), which is essential for early gametocyte development, was found to be under strong selection in *P*. *falciparum* populations in regions of different endemicity in West Africa [55]. In Cambodia, control efforts resulted in strong selection of the AP2-G homolog in *P*. *vivax* [56]. The present study, for the first time, found differences in the proportion of gametocytes among all parasites between regions of different transmission intensity.

## Conclusions

The probability to detect gametocytes was closely correlated to blood-stage parasitemia in different transmission settings. The vast majority of all infections with high gametocyte densities (as determined by RT-qPCR) could be diagnosed by microscopy. Pronounced age trends of gametocyte carriage in areas of moderate to high transmission was observed. Even though the contribution to transmission is influenced by vector exposure and transmission blocking immunity in addition to gametocyte prevalence and density, the age trends suggest that interventions to reduce transmission will have the greatest effect when targeted towards children. In contrast, in order to achieve elimination in low transmission settings individuals of all ages need to be protected from vector contact, which is often not the case [57].

## Supporting information

S1 Data Database. (TXT)

S1 Table Distribution of study participants by age group and by gender in each survey. (DOCX)

S2 Table Effect of hemoglobin levels on gametocyte positivity and density. (DOCX)

S3 Table Effect of multiple clone infection on gametocyte positivity and density. (DOCX)
