# Supplementary material for: Identification of the asymptomatic Plasmodium falciparum and Plasmodium vivax gametocyte reservoir under different transmission intensities
Source: PLoS Negl Trop Dis. 2021 Aug 27;15(8):e0009672. doi: 10.1371/journal.pntd.0009672 (PMC8428688; doi:10.1371/journal.pntd.0009672)
Supplement: S1 Table — (DOCX) [file pntd.0009672.s002.docx]

**Supplementary Table S1:** Distribution of study participants by age group and by gender in each survey

|  | **PNG 2010** | **PNG 2014** | **Brazil** | **Solomon Is.** | **Thailand** |
| --- | --- | --- | --- | --- | --- |
| **Age group** |  |  |  |  |  |
| 0-6 | 519 (24.9%) | 604 (24%) | 588 (14.4%) | 746 (21.3%) | 715 (16.6%) |
| 6-12 | 399 (19.2%) | 440 (17.5%) | 629 (15.4%) | 628 (17.9%) | 841 (19.5%) |
| 12-20 | 198 (9.5%) | 319 (12.7%) | 622 (15.2%) | 484 (13.8%) | 605 (14%) |
| >20 | 967 (46.4%) | 1154 (45.8%) | 2244 (55%) | 1643 (46.9%) | 2148 (49.8%) |
| Total | 2083 | 2517 | 4083 | 3501 | 4309 |
|  |  |  |  |  |  |
| **Gender** |  |  |  |  |  |
| 0 | 1131 (54.5%) | 1382 (54.9%) | 1945 (47.6%) | 1837 (52.5%) | 2222 (51.6%) |
| 1 | 945 (45.5%) | 1135 (45.1%) | 2138 (52.4%) | 1664 (47.5%) | 2087 (48.4%) |
| Total | 2076 | 2517 | 4083 | 3501 | 4309 |
