# Supplementary material for: Identification of the asymptomatic Plasmodium falciparum and Plasmodium vivax gametocyte reservoir under different transmission intensities
Source: PLoS Negl Trop Dis. 2021 Aug 27;15(8):e0009672. doi: 10.1371/journal.pntd.0009672 (PMC8428688; doi:10.1371/journal.pntd.0009672)
Supplement: S2 Table — (DOCX) [file pntd.0009672.s003.docx]

**Supplementary Table S2**: Effect of hemoglobin levels on gametocyte positivity and density (multivariable model including age and parasite density)

| **A) Gametocyte positivity** | |  |  |  |  |
| --- | --- | --- | --- | --- | --- |
|  | *P. falciparum* (n=562) | |  | *P. vivax* (n=1152) | |
|  |  |  |  |  |  |
|  | OR | P |  | OR | P |
| Hemoglobin [g/dL] | 0.94 | 0.31 |  | 0.98 | 0.727 |
| log10 Pf/Pv copies | 1.57 | <0.001 |  | 3.02 | <0.001 |
| age: 0-6 y (reference) | [reference] |  |  | [reference] |  |
| age: >6-12 y | 0.94 |  |  | 0.88 |  |
| age: >12-20 y | 0.53 | 0.0011 |  | 0.51 | 0.0198 |
| age: >20 y | 0.42 |  |  | 0.59 |  |
|  |  |  |  |  |  |
|  |  |  |  |  |  |
| **B) Gametocyte density** | |  |  |  |  |
|  | *P. falciparum* (n=557) | |  | *P. vivax* (n=717) | |
|  |  |  |  |  |  |
|  | Coef | P |  | Coef | P |
| Hemoglobin [g/dL] | -0.05 | 0.154 |  | -0.02 | 0.339 |
| log10 Pf/Pv copies | 0.29 | <0.001 |  | 0.5 | <0.001 |
| age: 0-6 y (reference) | [reference] |  |  | [reference] |  |
| age: >6-12 y | -0.52 |  |  | -0.09 |  |
| age: >12-20 y | -0.87 | 0 |  | -0.24 | 0.0088 |
| age: >20 y | -0.92 |  |  | -0.24 |  |
