# Supplementary material for: Identification of the asymptomatic Plasmodium falciparum and Plasmodium vivax gametocyte reservoir under different transmission intensities
Source: PLoS Negl Trop Dis. 2021 Aug 27;15(8):e0009672. doi: 10.1371/journal.pntd.0009672 (PMC8428688; doi:10.1371/journal.pntd.0009672)
Supplement: S3 Table — (DOCX) [file pntd.0009672.s004.docx]

**Supplementary Table S3**: Effect of multiple clone infection on gametocyte positivity and density (multivariable model including parasite density, as detectability of minority clones increases with increasing parasite density)

| **A) Gametocyte positivity** |  |  |  |  |  |
| --- | --- | --- | --- | --- | --- |
|  | *P. falciparum* (n=511) | |  | *P. vivax* (n=920) | |
|  |  |  |  |  |  |
|  | OR | P |  | OR | P |
| Multiclone infection | 1.52 | 0.1 |  | 1.34 | 0.072 |
| log10 Pf/Pv copies | 1.57 | <0.001 |  | 3.09 | <0.001 |
|  |  |  |  |  |  |
|  |  |  |  |  |  |
| **B) Gametocyte density** |  |  |  |  |  |
|  | *P. falciparum* (n=506) | |  | *P. vivax* (n=595) | |
|  |  |  |  |  |  |
|  | Coef | P |  | Coef | P |
| Multiclone infection | 0.15 | 0.324 |  | 0.07 | 0.353 |
| log10 Pf/Pv copies | 0.35 | <0.001 |  | 0.57 | <0.001 |
